# Extended disability leave and related costs among employed patients with versus without graft-versus-host disease following hematopoietic stem cell transplantation

**DOI:** 10.1007/s00520-025-09561-z

**Published:** 2025-06-05

**Authors:** Jingbo Yu, Valkal Bhatt, John Galvin, Krutika Jariwala-Parikh, Ellen Thiel

**Affiliations:** 1 Incyte Corporation, 1801 Augustine Cut-Off, Wilmington, DE 19803 USA; 2 Merative, 100 Phoenix Drive, Ann Arbor, MI USA

**Keywords:** Hematopoietic stem cell transplantation, Graft-versus-host disease, Disability, Costs, Productivity

## Abstract

**Purpose:**

Post–hematopoietic stem cell transplantation (HSCT) graft-versus-host disease (GVHD) is associated with considerable healthcare costs, and impaired productivity may represent an additional financial burden. This analysis quantified GVHD-associated indirect costs of productivity loss by comparing work-related disability leave claims between patients with versus without GVHD.

**Methods:**

Patients with claims for HSCT in the IBM^®^ MarketScan Commercial Database and the Health and Productivity Management Database between 1/1/09–12/31/19 and associated short- or long-term disability claims were included.

**Results:**

Of 354 patients with GVHD and 2629 patients without GVHD included in the analysis, a significantly greater percentage of patients with versus without GVHD had claims for short-term disability (GVHD, 43.5%; non-GVHD, 29.2%; *P* < 0.001), long-term disability (GVHD, 19.9%; non-GVHD, 6.3%; *P* < 0.001) or a combination of both (GVHD, 53.5%; non-GVHD, 30.5%; *P* < 0.001). Disability leaves were, on average, longer for patients with GVHD versus without GVHD (short-term, 103 vs 59 days, *P* < 0.001; long-term, 120 vs 92 days, *P* < 0.001). Mean indirect costs of workdays lost due to disability leave were significantly higher among those with versus without GVHD (short-term, $13,180 vs $7504, *P* < 0.001; long-term, $15,441 vs $11,850, *P* < 0.001). Mean all-cause healthcare costs were significantly higher among those with versus without GVHD (short-term leave: $295,241 vs $95,937, *P* < 0.001; long-term leave: $312,691 vs $94,285, *P* < 0.001).

**Conclusions:**

Most full-time employed patients with GVHD took disability leave, accounting for approximately half (127/261) of their total workdays, incurring higher indirect costs than patients without GVHD. Additionally, all-cause healthcare costs were threefold higher in patients with versus without GVHD.

## Introduction

Hematopoietic stem cell transplantation (HSCT) is a potentially curative treatment for several hematologic malignancies and disorders [1–3]. Despite improvements in HSCT procedures and use of routine prophylaxis, acute graft-versus-host disease (GVHD) develops in 30% to 60% of HSCT recipients, and chronic GVHD occurs in 30% to 40% of HSCT survivors within the first year of transplant, with increasing incidence thereafter [4–8].

GVHD post-HSCT is burdensome and may hinder patient functioning. Quality of life related to physical and mental function may be impaired [9, 10]. Returning to work after HSCT can be challenging and further complicated by the presence of GVHD [11–14]. A prospective longitudinal study found by questionnaire that 36% of patients had returned to work 1 year post-HSCT, and interviews identified treatment and side effects such as GVHD as a barrier to returning to work [11, 12]. In a self-reported health status survey, patients with mild, moderate, or chronic GVHD were less likely to report that they were working than HSCT survivors who never developed GVHD or had resolved GVHD, and symptom severity has been associated with the ability to work [13, 14]. Furthermore, GVHD is associated with increased healthcare resource utilization, including increases in hospital stays and visits. The resulting healthcare costs can be burdensome even for patients with insurance coverage [15–19].

Lost workdays and reduced productivity resulting from decreased function and prolonged medical follow-up may represent an additional financial burden; however, limited data examining this relationship exist. In the multicenter Chronic GVHD Consortium Response Measures Validation Study, a multi-institutional cohort of 190 patients with chronic GVHD in the United States and Canada who completed questionnaires covering financial concerns, income, employment, and insurance [18], lower mental and physical functioning, disability/unemployment, younger age, and lower income were associated with a greater financial burden. However, the magnitude of the cost attributed to lost productivity in GVHD was not described. The objectives of the present study were to quantify GVHD-associated costs and productivity loss by comparing work-related disability absences and associated direct and indirect costs among full-time employees with versus without GVHD.

## Methods

### Study design and patients

This retrospective study used data from the IBM^®^ MarketScan Commercial and Health and Productivity Management databases. These databases contain de-identified patient-level health data, productivity data (including workplace absence, short-term and long-term disability, and workers’ compensation), and medical and pharmacy claims. Data from across the United States are contributed by employers, commercial health insurance organizations, Medicare, and Medicaid. Records of short- and long-term disability are linked to medical and pharmacy claims from a subset of US employers that contribute data to the database. Short-term disability was defined as wage replacement insurance for injury or illness lasting ≤ 6 months; long-term disability was defined as wage replacement insurance for permanent disability lasting > 6 months. Patients aged ≥ 18 years at diagnosis with a claim for an allogeneic HSCT procedure between January 1, 2009, and December 31, 2019, were included in the analysis. Patients were grouped into GVHD or non-GVHD cohorts based on the presence or absence of ≥ 2 outpatient or ≥ 1 inpatient claim with a GVHD diagnosis following HSCT. The date of the first GVHD diagnosis was the index date. For patients without GVHD, an index date was randomly assigned to match the distribution of time (days) from HSCT to GVHD in the GVHD cohort. Continuous enrollment ≥ 6 months pre-index and ≥ 12 months post-index was required. Only patients with data pertaining to either short- or long-term disability leave claims (those who met eligibility criteria based on the Health and Productivity Management Database) during the 12-month post-index follow-up period were included.

### Statistical analyses

Data on the frequency and duration of short- and long-term disability leave were summarized using descriptive statistics. Costs of disability leave were calculated by multiplying the number of days absent by the median daily wage (from 2019 US Bureau of Labor Statistics) and adjusted to 70% wage, which is typical of disability pay rates. Total direct medical costs (inpatient admissions, outpatient medical services, emergency room visits, and outpatient pharmacy costs) were quantified during the 12 months of follow-up and summarized using descriptive statistics. Costs were based on paid amounts of claims, including insurer and health plan payments, and the patient-paid amount. GVHD and non-GVHD cohorts were compared via *t* tests and chi-square tests.

## Results

### Patient demographics and clinical characteristics

Of the 6926 patients with at least 1 claim for HSCT, 671 met the criteria for GVHD and 6187 for non-GVHD (Table 1), with 354 (52.8%) patients with GVHD and 2629 (42.5%) without GVHD meeting all study criteria. Of these, 177 (50.0%) patients with GVHD and 1507 (57.3%) without GVHD were included in the short-term disability analysis. The long-term disability analysis included 166 (46.9%) patients with GVHD and 1498 (57.0%) without GVHD. Both short- and long-term data were available from 144 (40.7%) patients with GVHD and 1292 (49.1%) without GVHD. For both the short- and long-term disability analyses, the cohort of patients with GVHD had a greater percentage of men compared with the cohort without GVHD (short-term, 71.8% vs 49.1%; long-term, 71.7% vs 47.8%; both *P* < 0.001; Table 2). Other demographics were similar between cohorts of patients with and without GVHD, including median age, which was approximately 50 years for all cohorts. Median (range) time from HSCT to first GVHD diagnosis was 79.0 (1–1305) and 78.0 (1–1121) days, respectively, for patients with GVHD with available short- and long-term disability data. Approximately 10% of patients with GVHD in both the short- and long-term disability analyses had only acute GVHD, more than one-third (37.3%–39.0%) had only chronic GVHD, and half (50.8%–52.4%) developed both acute and chronic GVHD (Table 2).

**Table 1:** Patient Attrition

Criteria	GVHD		Non-GVHD	
	N	%^a^	N	%^a^
≥ 1 inpatient or ≥ 2 nondiagnostic outpatient medical claims with a diagnosis for GVHD any time after date of HSCT^b^	671	9.7^c^	—	—
No medical claim with a diagnosis of GVHD any time after date of HSCT	—	—	6187	89.3^c^
≥ 18 years old at index date	671	100	5061	81.8
≥ 6 months of continuous enrollment before index date	634	94.5	4801	77.6
≥ 12 months of continuous enrollment after index date	354	52.8	2629	42.5
≥ 12 months of continuous eligibility after index date and whose employers contributed disability leave claims data for that period:	354	52.8	2629	42.5
With short-term disability leave claim^d^	177	50.0	1507	57.3
With long-term disability leave claim^d^	166	46.9	1498	57.0
With short- and long-term disability leave claims^d^	144	40.7	1292	49.1

GVHD, graft-versus-host disease; HSCT, hematopoietic stem cell transplantation.

^a^Percentages based on number of patients with GVHD or non-GVHD, unless indicated.

^b^Patients without a medical claim for GVHD were selected from a simulated time period based on the mean/median distribution of days from the date of HSCT to the date of GVHD in the GVHD cohort.

^c^Percentage based on the 6926 patients with at least 1 claim for an HSCT procedure between January 1, 2009, and December 31, 2019.

^d^Percentages listed in these rows are based on patients with ≥ 12 months of continuous eligibility after index date and whose employers contributed disability leave claims data (GVHD, *N * = 354; non-GVHD, *N * = 2629). Patients with a short-term and long-term disability claim are included in the short-term, long-term, and short- and long-term categories; patients with only a short-term or long-term disability leave claim will be included in the corresponding category only.

**Table 2:** Baseline Patient Demographics and Clinical Characteristics

Characteristic	Short-Term Disability Cohorts			Long-Term Disability Cohorts		
	GVHD (*n* = 177)	Non-GVHD (*n* = 1507)	*P* Value	GVHD (*n* = 166)	Non-GVHD (*n* = 1498)	*P* Value
Age, median (range), years	50.0 (23–63)	49.0 (21–63)	–	50.5 (24–63)	49.0 (21–63)	–
Men, n (%)	127 (71.8)	740 (49.1)	< 0.001	119 (71.7)	716 (47.8)	< 0.001
Geographic region,^a^ n (%)						
Northeast	28 (15.8)	289 (19.2)	0.28	22 (13.3)	268 (17.9)	0.14
North central	40 (22.6)	329 (21.8)	0.82	44 (26.5)	358 (23.9)	0.46
South	65 (36.7)	650 (43.1)	0.10	59 (35.5)	636 (42.5)	0.09
West	44 (24.9)	234 (15.5)	< 0.01	41 (24.7)	231 (15.4)	< 0.01
Family size, n (%)						
1	43 (24.3)	453 (30.1)	0.11	40 (24.1)	462 (30.8)	0.07
2	31 (17.5)	321 (21.3)	0.24	29 (17.5)	315 (21.0)	0.28
3	37 (20.9)	266 (17.7)	0.29	35 (21.1)	260 (17.4)	0.23
≥ 4	66 (37.3)	467 (31.0)	0.09	62 (37.3)	461 (30.8)	0.08
CCI, median (range)	2.0 (0–13)	2.0 (0–15)	–	2.0 (0–13)	2.0 (0–15)	–
Time from HSCT to first GVHD diagnosis, median (range), days	79.0 (1–1305)	N/A	–	78.0 (1–1121)	N/A	–
GVHD type, n (%)						
Acute only	18 (10.2)	N/A	–	17 (10.2)	N/A	–
Chronic only	69 (39.0)	N/A	–	62 (37.3)	N/A	–
Acute and chronic	90 (50.8)	N/A	–	87 (52.4)	N/A	–

*CCI* Charlson Comorbidity Index, *GVHD* graft-versus-host disease, *HSCT* hematopoietic stem cell transplantation, *N/A* not applicable

^a^Region was unknown for 5 patients each in the short- and long-term disability cohorts (all non-GVHD)

### Short- and long-term disability leave

**Fig. 1:**
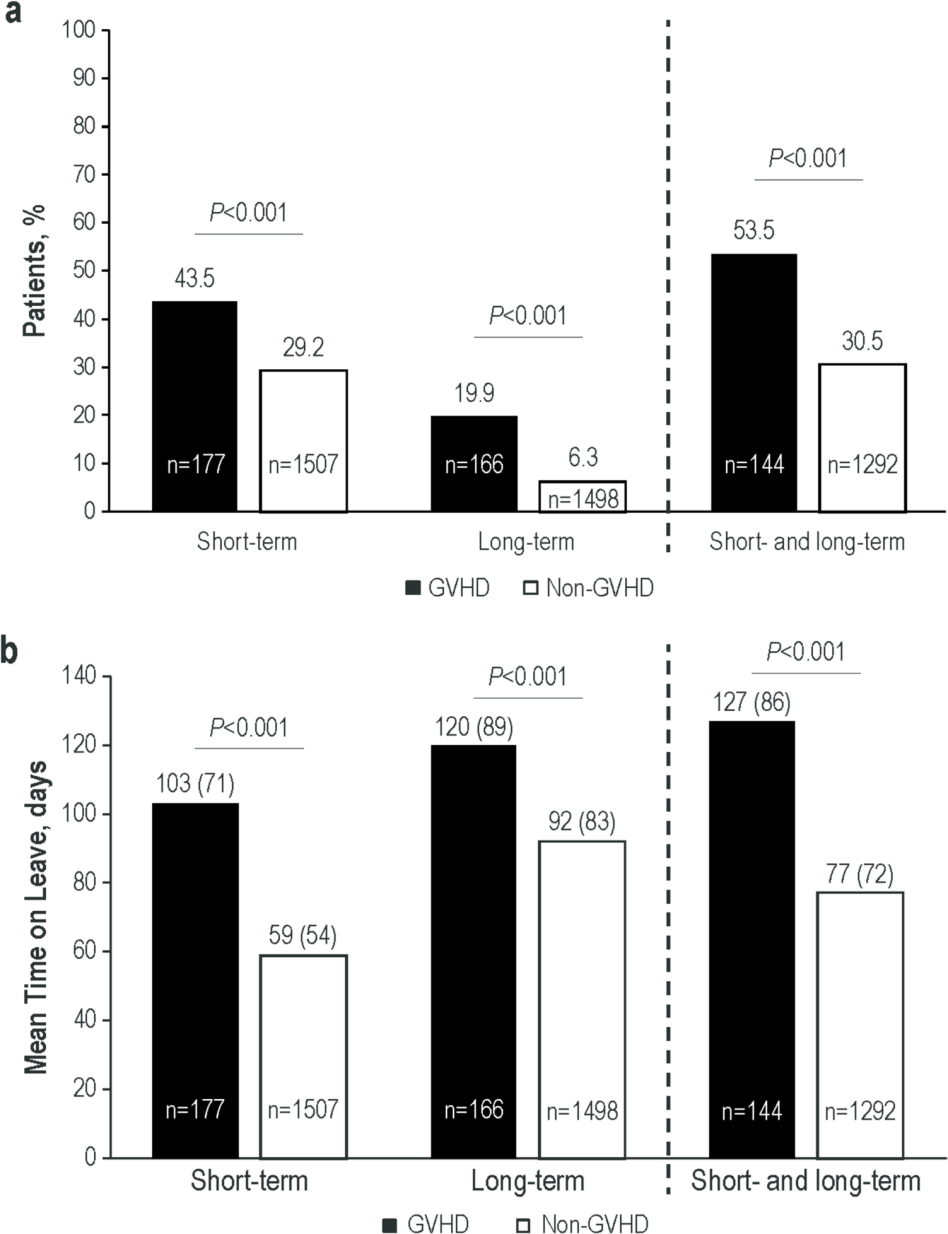
(**a**) Patients With ≥ 1 Short- or Long-Term Disability Leave During 12-Month Follow-Up and (**b**) Mean (SD) Time on Leave. GVHD, graft-versus-host disease

Short-term disability leave was taken by a significantly higher percentage of patients with versus without GVHD (43.5% vs 29.2%, *P* < 0.001; Fig. 1a), as was long-term disability leave (19.9% vs 6.3%, *P* < 0.001). Overall, the majority of patients with GVHD had both a short- and long-term disability leave claim, a significantly higher percentage than was observed for patients without GVHD (53.5% vs 30.5%, *P* < 0.001). Among those with a disability claim, the leaves were also longer among patients with versus without GVHD, with mean short-term disability absences being nearly twice as long (103 vs 59 days, *P* < 0.001) and mean long-term disability leaves persisting approximately 1 month longer (120 vs 92 days, *P* < 0.001) for patients with versus without GVHD (Fig. 1b). For patients with both short- and long-term disability claims, mean leave time for patients with GVHD was 127 days, nearly half of the 261 workdays in the 12-month follow-up period (assuming a 5-day work week). In contrast, among patients with short- and long-term disability claims without GVHD, mean leave time was 77 days (*P* < 0.001).

### Costs

**Fig. 2:**
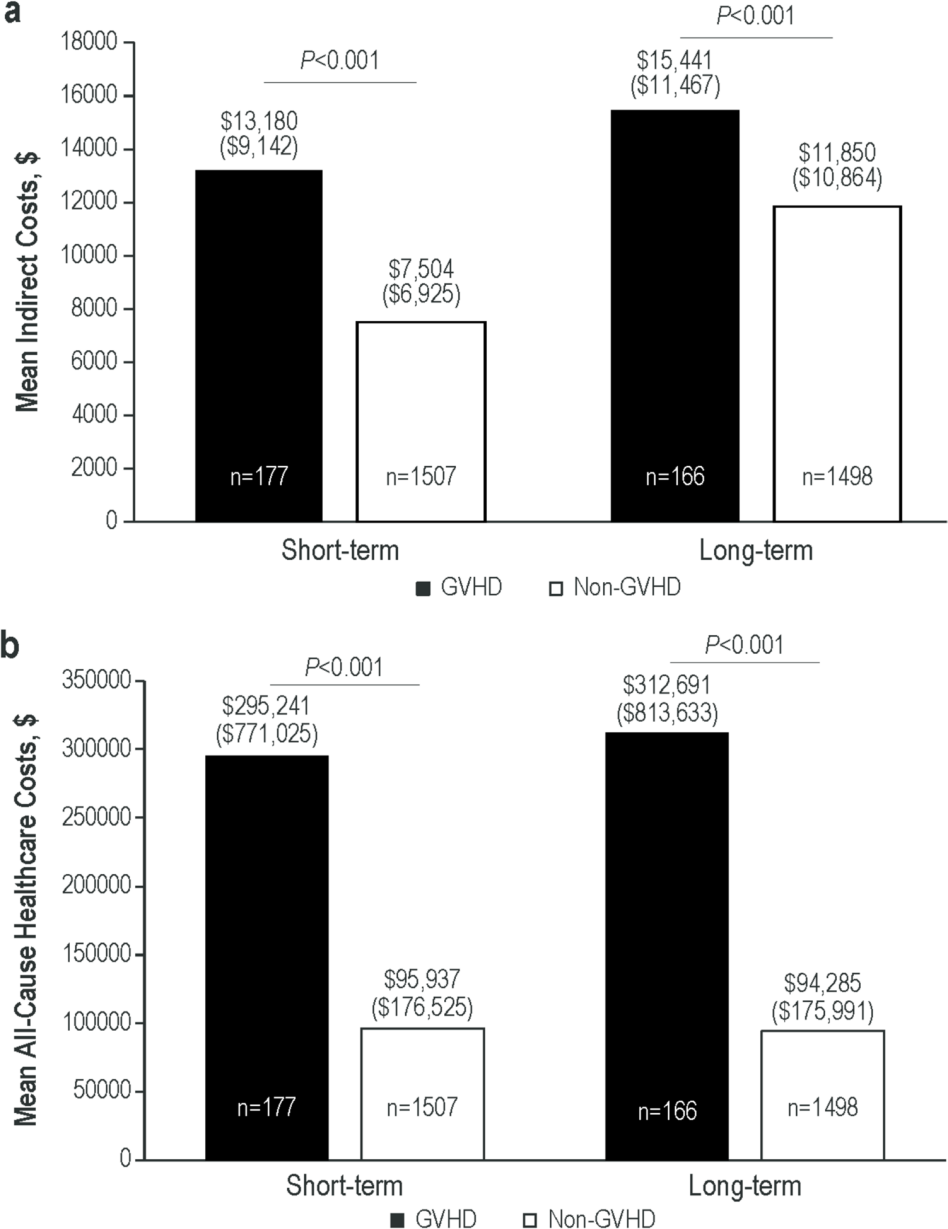
Mean (SD) (**a**) Indirect Costs due to Missed Workdays on Disability Among Patients With Short- and Long-Term Disability Leave and (**b**) Total Direct All-Cause Healthcare Costs During 12-Month Follow-Up Among Patients With Short- and Long-Term Disability Leave. GVHD, graft-versus-host disease

Mean indirect costs of workdays lost due to disability leave during the 12-month follow-up period were significantly higher among patients with versus without GVHD (Fig. 2a). This was most marked among patients in the short-term disability analysis cohort ($13,180 vs $7504, *P* < 0.001). However, patients with GVHD also had approximately 30% higher mean indirect costs related to long-term disability leave compared with those without GVHD ($15,441 vs $11,850, *P* < 0.001).

Mean all-cause healthcare costs were significantly higher among patients with versus without GVHD (Fig. 2b). These costs were approximately 3 times higher among patients with versus without GVHD in both the short-term disability ($295,241 vs $95,937, *P* < 0.001) and long-term disability cohorts ($312,691 vs $94,285, *P* < 0.001).

## Discussion

This retrospective analysis of HSCT recipients demonstrated that patients with GVHD were more likely to require a disability leave from work (either short- or long-term disability), with longer absences, compared with patients without GVHD. Further, mean indirect costs due to workdays lost and all-cause healthcare costs were higher among patients with GVHD, regardless of whether disability leaves were short- or long-term.

The results presented here are consistent with previously published studies that demonstrate high healthcare costs associated with GVHD [15, 16]. A retrospective single-center analysis of 187 HSCT recipients in the United Kingdom found that mean readmission costs were more than double for patients who developed acute GVHD compared with those for patients without GVHD (£28,860 vs £13,405 [based on the 2010 hospital tariff and the 2009–2010 National Health Services Reference Costs]) [15]. A retrospective review of a large US hospital database examined payer data for HSCT recipients between 2011–2016. Patients with acute GVHD (*n* = 906) had higher median costs of initial hospital stays ($153,849) and greater median readmission costs during the first 100 days following HSCT ($18,348) than patients without GVHD (*n* = 1529; $97,417 and $13,361, respectively). The increased costs of acute GVHD remained apparent after controlling for the increased length of stay also associated with acute GVHD, with a median cost per day approximately $200 higher for patients with versus without GVHD ($2725 vs $2507) [16]. These previous studies focused on inpatient costs associated with GVHD. We expanded on these findings by also considering the cost of outpatient office visits, with mean all-cause healthcare costs being 3 times higher in patients with versus without GVHD.

Other studies analyzing socioeconomic domains have also touched on the impact of GVHD on patients’ work or school status. In the prospective, multicenter Chronic GVHD Consortium Response Measures Validation study, two-thirds of the 190 questionnaire respondents with chronic GVHD reported financial burdens, including costs of medication and ongoing treatment, as well as an inability to return to work [18]. However, unlike the present study, financial burden from healthcare costs or lost productivity was not analyzed. In another prospective, real-world analysis, as part of a long-term follow-up research program, quality-of-life domains among HSCT survivors were assessed by questionnaire [13]. Among 1377 respondents, patients with chronic GVHD were less likely to report being employed or in school (61%–73%) versus 85% who had never been diagnosed with chronic GVHD and 88% with resolved disease. The prospective, multicenter Chronic GVHD Consortium Improving Outcomes Assessment study also analyzed effects on ability to attend work or school, reporting that among 421 patients with chronic GVHD, 47% were unable to attend work or school [14]. An inability to work was the only socioeconomic variable included in that analysis that was associated with higher overall mortality. Inability to work was also associated with worsening of patient-reported GVHD symptoms and poorer quality of life, but not with physician-rated severity of disease. Patients with the ability to work had a lower mortality risk relative to those unable to work. Patients who were homemakers, retired, unemployed, or not looking for work had a mortality risk comparable to those unable to work. However, whether work status was a measure of socioeconomic status, a reflection of patient well-being, or a combination of both was undetermined. A longitudinal, prospective single-center study following 237 HSCT recipients analyzed factors associated with full-time sick leave within 1 year following transplant. Having a diagnosis of chronic GVHD was associated with taking full-time sick leave (odds ratio, 3.07); a multivariable analysis confirmed chronic GVHD as 1 of 2 factors independently associated with taking full-time sick leave [20]. These studies contribute to our understanding of GVHD by describing work status among patients with GVHD but do not provide the costs of the indicated lost productivity. The present study builds on these previous findings, contributing to a more complete description of the financial burden of GVHD by quantifying lost productivity costs associated with GVHD in HSCT recipients.

In the current analyses, patients with GVHD were more likely to be men. Donor sex as a GVHD risk factor is well documented, with a described association between female donor and increased risk of GVHD for both male and female recipients [21]. Consistent with the current study, being a male recipient has previously been shown to be associated with the development of GVHD following HSCT. Retrospective analysis of data from the European Society for Blood and Marrow Transplantation (EBMT) showed that the percentage of female HSCT recipients who did not develop GVHD was higher than among male recipients [21], albeit to a lesser degree than the sex difference observed here.

Analyses of sex differences and return to work in conditions other than GVHD, including HSCT, have shown women to be less likely to return to work and more likely to resign from work [11, 22, 23], possibly due to men being more likely to be the primary wage earner in a family and thus feeling more pressure to return to work. However, these studies did not conclude if the relationship between patient sex and returning to work was due to health-related factors or different factors, such as work intentions or social context. Health-related factors have been linked to recurrent sick leave following HSCT, with the primary causes for recurrent sick leave reported as physical issues, including GVHD, infection, and hospital readmission [24]. Interestingly, a survey of patients following HSCT found that of those who had returned to work, 45% adjusted their work practices, including flexible or part-time working, or working from home [25]; such adjustments may go some way to minimizing the health-related factors affecting patients’ return to work and recurrent sick leave. Together, these findings suggest that support to address health-related factors may increase the number of patients returning to work after HSCT, especially among women, and reduce rates of recurrent sick leave.

Limitations of this study include those inherent to retrospective analyses, such as the potential for inaccurate or incomplete data entries. In particular, the analysis included acute or chronic GVHD diagnoses based on medical claims and were not verified by the study investigators. Additional limitations include the requirement for ≥ 12 months of continuous enrollment after GVHD diagnosis and exclusion of patients who died early during the course of disease; only patients who survived GVHD for at least a year were included in the analysis. This study used databases of individuals in the United States with employer-provided short- and long-term disability leave coverage for at least 18 months; therefore, the results may not be generalizable to people with other types of insurance or without health insurance coverage. People without employer-provided insurance are more likely to be minorities and have higher rates of poverty [26]. Furthermore, different types of insurance, such as employer-provided, exchange-based, or public insurance, may negotiate variable rates with healthcare providers [27]. Disability leave provision may also vary across different types and sizes of employers [28]; data here are predominantly from a convenience sample of large employers, with smaller employers possibly underrepresented. Therefore, the estimates of direct and indirect costs of GVHD in this study may not reflect those paid by other types of plans or for all employees. Further studies to assess costs in other demographics are needed.

Strengths include that this study examined the real-world costs of GVHD clinical care among the largest segment of insured US healthcare users across the country [26]. Data originated from a large number of employers and pertained to a large sample of patients who underwent HSCT. The study design directly linked patient data with short- and long-term disability claims data to accurately assess healthcare costs and disability leave to estimate the cost of lost workdays.

In summary, findings from this retrospective analysis of medical claims reinforce the financially burdensome nature of GVHD, highlighting its association with lost workdays and higher indirect and overall healthcare costs. By demonstrating that employed patients with versus without GVHD post-HSCT incurred higher indirect costs, the results suggest that previous descriptions of the financial burden in GVHD that were limited to direct costs may have been underestimates. Prevention and effective treatment of GVHD could play an important role in reducing work disability and overall financial burden for patients following HSCT.

## Data Availability

Access to individual patient-level data is not available for this study. Information on Incyte’s clinical trial data sharing policy and instructions for submitting clinical trial data requests are available at: https://www.incyte.com/Portals/0/Assets/Compliance%20and%20Transparency/clinical-trial-data-sharing.pdf.
